# Lymph Node Ratio as a Prognostic Marker in Rectal Cancer Survival: A Systematic Review and Meta-Analysis

**DOI:** 10.7759/cureus.8047

**Published:** 2020-05-10

**Authors:** Uday Karjol, Pavan Jonnada, Ajay Chandranath, Sushma Cherukuru

**Affiliations:** 1 Surgical Oncology, Kidwai Memorial Institute of Oncology, Bangalore, IND; 2 Pathology, AmPath Laboratories, Hyderabad, IND

**Keywords:** lymph node, ratio, rectal cancer, disease free survival, overall survival, systematic review and meta-analysis

## Abstract

Introduction

The lymph node ratio (LNR) is defined as the ratio of the number of positive lymph nodes to the total number of nodes retrieved. LNR has recently emerged as a prognostic factor in rectal cancer. The objective of our study was to pool eligible studies to elucidate the prognostic role of LNR on overall survival (OS) and disease-free survival (DFS) in rectal cancer patients using a meta-analysis.

Methods

A systematic database search was performed in MEDLINE and Embase for relevant studies that reported LNR in rectal cancer. Two authors independently screened the relevant articles for selection and data extraction. As a result, a list of such studies and references, published in English up to December 2019, was obtained, and a total of 4,486 node-positive patients in 18 studies were included in this meta-analysis. RevMan software 5.3 (Cochrane Collaboration, the Nordic Cochrane Centre, Copenhagen) was used for conducting all statistical analyses.

Results

A higher LNR was significantly correlated with worse OS [hazard ratio (HR): 2.60; 95% confidence interval (CI): 2.21-3.06; p≤.00001] and DFS (HR: 2.43; 95% CI: 2.11-2.80; p≤.00001) in node-positive rectal cancer patients. Besides, LNR is an independent predictive and prognostic marker of OS and DFS (HR: 2.52; 95% CI: 2.17-2.94; p≤.00001 with I^2^=0%; p=.32 and HR: 2.63; 95% CI: 2.17-3.18; p≤.00001 with I^2^=0%; p=.63 respectively, irrespective of lymph nodal harvest).

Conclusions

Our present study demonstrates that LNR is an independent predictor of survival in rectal cancer. LNR should be considered as a parameter in future oncological staging systems. Further well-designed randomized control trials to prospectively assess LNR as an independent predictor of rectal cancer survival are necessary before its application in daily practice.

## Introduction

Colorectal cancer is the third most common cancer and the second leading cause of cancer deaths globally [1]. Lymph node metastasis is considered as an important factor for predicting overall survival (OS) and disease-free survival (DFS) in non-metastatic rectal cancer [2]. Lymph node status is an essential factor in determining the need for adjuvant chemotherapy after surgical resection. The assessment of lymph node metastasis in colorectal cancer is accomplished by the tumor node metastasis (TNM) staging system. This system stages lymph node involvement according to the absolute number of the positive regional lymph nodes and recommends harvesting of at least 12 nodes. In the current staging system, rectal cancer with regional lymph node metastasis is classified as stage three, which has additional treatment adjuncts [3]. However, many reports have demonstrated a decrease in the total number of harvested lymph nodes following neoadjuvant therapy. This can lead to an underestimation of nodal staging, which may lead to false-negative nodal disease or lower nodal stage [4].

The lymph node ratio (LNR) is defined as the ratio of metastatic to the total number of harvested lymph nodes, and it has emerged as an indicator of cancer-specific survival in recent years. Berger et al. have analyzed the prognostic significance of LNR in colon cancer. They observed the data from the intergroup trial-0089 of adjuvant chemotherapy for stage II and III colon cancer patients and concluded that LNR is a significant factor for DFS, OS, and cancer-specific survival in patients in whom more than 10 lymph nodes were retrieved [5]. This highlighted the importance of adequate lymph node retrieval and LNR. In the present study, using a meta-analysis, we aimed to clarify the prognostic role of LNR in patients with node-positive rectal cancer. To that end, we examined the relationship of LNR with OS and DFS in such patients.

## Materials and methods

Search strategy

We performed a systematic literature search on MEDLINE, Embase, and Google Scholar databases for articles published before January 2020 using the following strategy: articles were searched using Medical Education Subject Headings (MeSH) keywords "lymph node" AND "ratio" AND "rectal cancer" OR "rectal carcinoma" AND "node-positive" OR "metastatic lymph node." Preferred Reporting Items for Systematic Reviews and Meta-Analyses (PRISMA) guidelines were followed for searching and reporting of articles.

Study selection

All studies that reported an association of LNR with OS and DFS for rectal cancer patients were identified by a comprehensive computer-based search. Two authors (PJ and UK) independently assessed titles and abstracts for eligibility. We scanned the reference lists of articles for similar additional articles. All the screened articles were assessed for eligibility, and any disagreement was resolved through discussion. We included studies in the meta-analysis if the following criteria were met: studies that were published in English, studies that were clinical trials, studies that compared the survival of rectal cancer based on LNR, and studies that included quantitative outcome data after multivariate analysis [hazard ratio (HR) for OS and DFS]. The exclusion criteria were as follows: inability to extract data from the published results; studies containing republished data; publications in the form of editorials, comments, review articles, meeting abstracts, or those which excluded reported outcomes.

Data extraction

Two authors (PJ and UK) independently extracted relevant data from the screened full-text articles. For each study that fulfilled the criteria for inclusion, the data extracted include the following: the basic characteristics of the study including the name of the first author, year of publication, study setting, design of the study, duration of the study, data sources, and multivariate adjustments; the basic patient characteristics including age, gender, stage, treatment, and survival periods; comparative outcomes, including HR for OS, DFS, and recurrence on different LNR subgroups.

Quality assessment

Two authors (PJ and AC) independently appraised the quality of each included study using the Newcastle-Ottawa scale. The details of the included studies are shown in Table 1 [6-23]. A study was considered of poor quality if it did not meet more than one criterion in the selection domain, if there was no score in the compatibility domain, and if it did not meet more than one of the criteria in the outcome domain. Any disagreements between reviewers were resolved by consensus.

**Table 1:** Characteristics of the included studies NACRT: neoadjuvant chemoradiotherapy; NOS: Newcastle-Ottawa score; DFS: disease-free survival; NA: not available; OS: overall survival; PCS: prospective cohort study; RCS: retrospective cohort study

Author name	Year	Study design	Sample size	NACRT	No. of average nodes	Endpoints		Median follow-up (months)	NOS	LNR stratification			
Peng et al. [6]	2008	RCS	318	No	12	OS	DFS	41	7	<0.14	0.14-0.49	>0.49	-
Kim et al. [7]	2009	RCS	421	No	17	OS	-	53	7	<0.1	<0.2	<0.4	>0.4
Dekker et al. [8]	2010	RCS	605	Yes	9	OS	DFS	120	7	<0.6	>0.6	-	-
Kang et al. [9]	2011	RCS	75	Yes	18	OS	-	35.1	7	<0.143	>0.143	-	-
Kobayashi et al. [10]	2011	RCS	452	No	17	OS	-	NA	8	<0.04	0.04-0.07	0.08-0.15	0.15-1
Allaix et al. [11]	2012	PCS	129	Yes	12	OS	DFS	122	7	0.01-0.25	>0.25	-	-
Lee et al. [12]	2012	PCS	519	Yes	15	OS	DFS	52	7	<0.15	0.16-0.3	>0.3	-
Madobouly et al. [13]	2013	PCS	115	Yes	12	OS	DFS	37	6	<0.375	>0.375	-	-
La Torre et al. [14]	2013	PCS	508	Yes	15	OS	DFS	50	8	<0.2	>0.2	-	-
Nadoshan et al. [15]	2013	PCS	128	Yes	10	OS	DFS	39	8	<0.2	>0.2	-	-
Junginger et al. [16]	2014	PCS	237	Yes	NA	OS	DFS	55	8	<0.1	<0.2	<0.3	>0.3
Zeng et al. [17]	2014	PCS	131	Yes	14	OS	DFS	49	8	<0.2	>0.2	-	-
Koo et al. [18]	2015	RCS	125	Yes	17	OS	DFS	55	8	<0.15	>0.15	-	-
Park et al. [19]	2015	RCS	967	Yes	16.5	-	DFS	40	8	<0.25	>0.25	-	-
Leonard et al. [20]	2016	RCS	357	Yes	13	OS	DFS	NA	7	<0.2	>0.2	-	-
Zuo et al. [21]	2016	RCS	264	Yes	11	OS	DFS	45	7	<0.2	>0.2	-	-
Fritzmann J et al. [22]	2018	PCS	630	Yes	15	OS	-	36.1	7	<0.01-0.17	0.18-0.41	0.42-0.69	>0.69
Chen et al. [23]	2018	RCS	133	Yes	12	OS	DFS	40	7	<0.15	>0.15	-	-

Statistical analysis

The statistical analysis was performed using RevMan software, version 5.3 (Cochrane Collaboration, the Nordic Cochrane Centre, Copenhagen). Continuous variables were analyzed by the HR, and 95% CI was recorded. Heterogeneity was assessed using χ2 and I^2^ tests. I^2 ^of 0-40, 30-60, 50-70, and >75% represent low, moderate, substantial, and considerable heterogeneity, respectively. Studies with a p-value of <.1 and I^2^ indicated substantial heterogeneity. A random-effects model was used to estimate the pooled HR if significant heterogeneity existed in the fixed-effects model. Otherwise, the fixed-effects model was used with p>.10 and I^2^<25%. The z-test was used to determine the pooled HR, and the significance was set to reject the null hypothesis at p<.05. Funnel plots were undertaken to investigate possible bias.

## Results

Studies included

**Figure 1:**
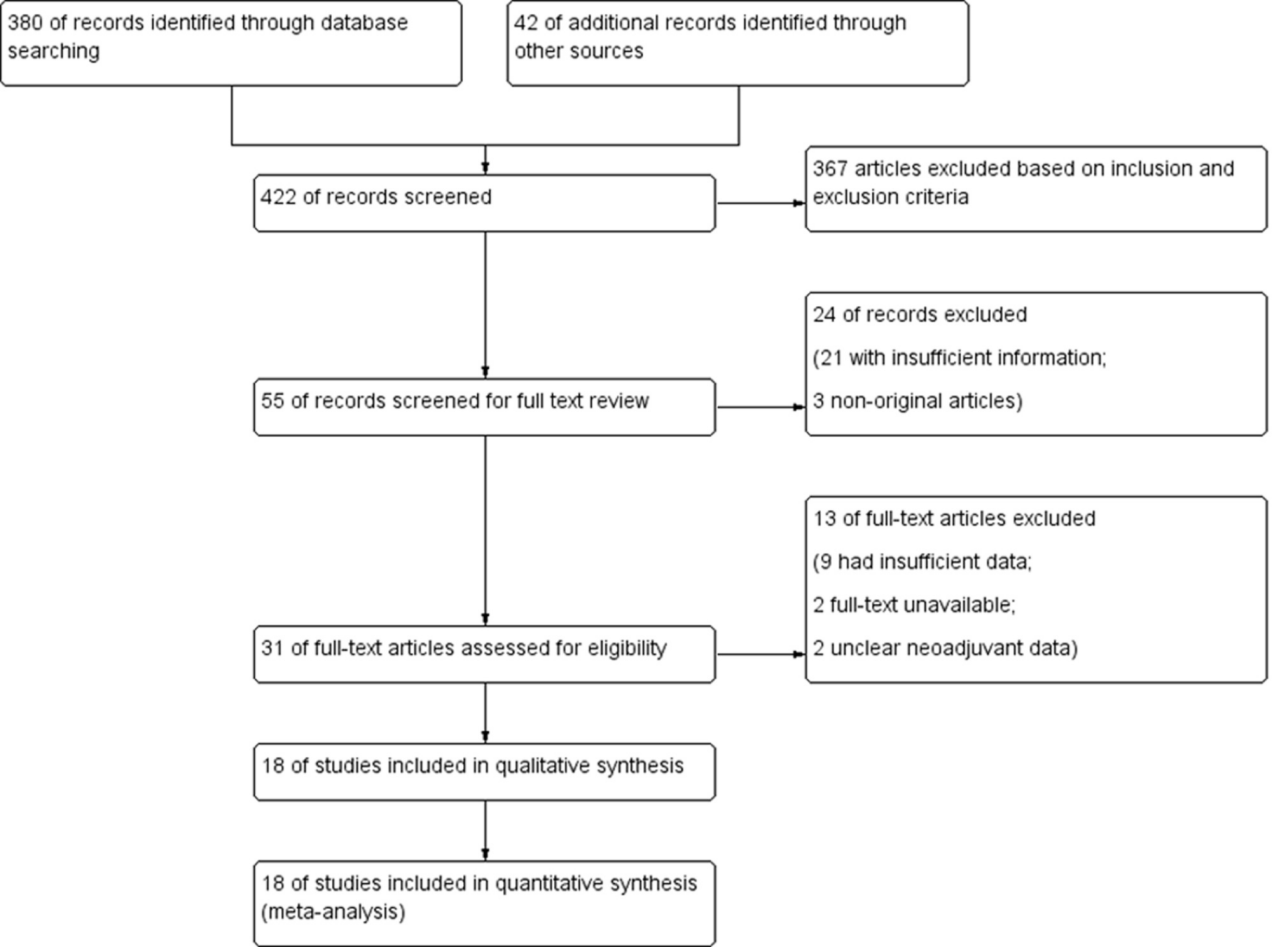
PRISMA flow chart showing study selection PRISMA: Preferred Reporting Items for Systematic Reviews and Meta-Analyses

A total of 422 potentially relevant articles were identified with our predefined search strategy. Based on inclusion and exclusion criteria and following the screening of titles and abstracts, 367 studies were excluded. After excluding duplicates, the reviewers identified 31 studies for an extensive review. Of these, 18 studies were entered into meta-analysis after the exclusion of 24 studies (Figure 1). The quality of articles as assessed by the Newcastle-Ottawa score was by and large acceptable. The main characteristics of the included studies are provided in Table 1.

Meta-analysis results

**Figure 2:**
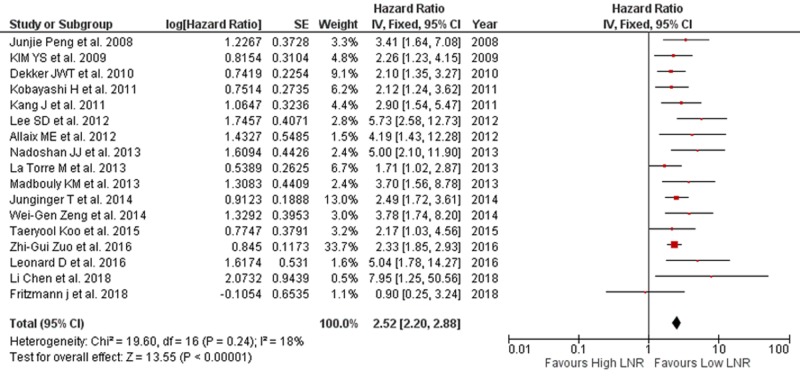
Forest plot showing LNR and OS OS: overall survival; LNR: lymph node ratio; SE: standard error; CI: confidence interval

The estimated mean number of harvested lymph nodes was 12.9 ±1.03 in cases overall. In the present study, we performed the meta-analysis and examined the effect of LNR on OS and DFS. OS, as the primary outcome, was extracted from 17 studies with available data. A pooled HR and its 95% confidence interval (CI) were calculated with a fixed model for OS (Figure 2) [6,8,11-21,23]. The result showed that high LNR predicts poor OS. The pooled HR was 2.52 (95% CI: 2.20-2.88) for OS with a statistically significant p-value of <.00001. Insignificant heterogeneity was found (I^2^=18% and p=.24) on the fixed-effects model. Among these studies, 11 reported LNR with a single cut-off value and pooled HR of 2.64 (95% CI: 2.13-3.27; p<.00001). Insignificant heterogeneity was found (I^2^=11% and p=.34) on the fixed-effects model. Among these studies, five used 0.2, two used 0.15, one used 0.14, one used 0.375, and one study used 0.6 and reported a pooled HR of 2.50 (95% CI: 2.13-2.94; p<.00001) with little heterogeneity (I^2^=16%, p=.30). Two studies reported LNR with two cut-off values subdividing the patients into low risk, medium risk, and high-risk LNR groups, and they reported pooled HR of 4.32 (95% CI: 2.52-7.41; p<.00001). Minor heterogeneity was found (I^2^=0% and p=.35). Four studies reported three cut-off values, and they reported polled HR of 2.25 (95% CI: 1.72-2.93; p<.00001), with minimal heterogeneity (I^2^=0%, p=.51). Neoadjuvant therapy was given before surgery in 14 studies, and they reported pooled HR of 2.70 (95% CI: 2.18-3.34) with I^2^=30% and p=.14. Thirteen studies reported retrieval of more than 12 nodes with pooled HR of 2.78 (95% CI: 2.18-3.55) with I^2^=24% and p=.21, and three studies reported retrieval of 12 or more nodes with pooled HR of 2.37 (95% CI: 1.95-2.89), as shown in Table 2. It was demonstrated that reported retrieval of more than 12 nodes and less than 12 nodes showed an overall pooled HR of 2.52 (95% CI: 2.17-2.94; p≤.00001) with I^2^=0% and p=.32.

**Table 2:** Data for LNR and OS CI: confidence interval; CTRT: chemoradiation; HR: hazard ratio; LNR: lymph node ratio; OS: overall survival

Subgroups		Pooled estimates				Heterogeneity		
		No.of studies	HR	95% CI	P-value	Model	I^2^%	P-value
Overall		17	2.52	2.20-2.88	.00001	Fixed	18%	0.24
No.of nodes	<12	3	2.37	1.95-2.89	.00001	Fixed	37%	0.21
	≥12	13	2.78	2.18-3.55	.00001	Fixed	24%	0.21
CTRT	Yes	14	2.70	2.18-3.34	.00001	Fixed	30%	0.14
	No	3	2.50	1.88-3.31	.00001	Fixed	0%	0.59
LNR cut-off	0.1	2	3.25	1.74-6.09	.00001	Fixed	2%	0.31
	0.2	6	2.61	1.93-3.54	.00001	Random	36%	0.17
	0.3	2	4.69	2.61-8.42	.00001	Fixed	0%	0.47
	0.6	2	1.92	1.26-2.91	.002	Fixed	33%	0.22

**Figure 3:**
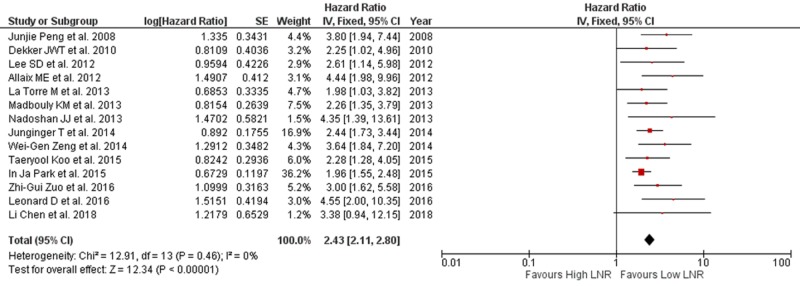
Forest plot showing LNR and DFS DFS: disease-free survival; LNR: lymph node ratio; SE: standard error; CI: confidence interval

DFS, as the primary outcome, was extracted from 14 studies with available data. A pooled HR and its 95% CI were calculated with a fixed model for OS (Figure 3). The result showed that low LNR is associated with improved DFS, and high LNR predicts poor DFS. The pooled HR was 2.43 (95% CI: 2.11-2.80) for DFS with a statistically significant p-value of <.00001. Insignificant heterogeneity was found (I^2^=0% and p=.46) on the fixed-effects model. Among these studies, nine reported LNR with a single cut-off value and pooled HR of 2.86 (95% CI: 2.26-3.62; p<.00001). Insignificant heterogeneity was found (I^2^=0% and p=.64) on the fixed-effects model. Two studies reported LNR with two cut-off values subdividing the patients into low risk, moderate risk, and high-risk LNR groups, and they reported pooled HR of 3.27 (95% CI: 1.94-5.52; p<.00001). Minor heterogeneity was found (I^2^=0% and p=.49). Neoadjuvant therapy was given before surgery in 14 studies, and they reported pooled HR of 2.79 (95% CI: 2.25-3.47) with I^2^=0% and p=.49. Thirteen studies reported retrieval of more than 12 nodes with pooled HR of 2.38 (95% CI: 2.01-2.80) with I^2^=21% and p=.25, and three studies reported retrieval of 12 or more nodes with pooled HR of 2.90 (95% CI: 1.85-4.54), as shown in Table 3. It was demonstrated that reported retrieval of more than 12 nodes and less than 12 nodes showed an overall pooled HR of 2.63 (95% CI: 2.17-3.18; p≤.00001) with I^2^=0% and p=.63.

**Table 3:** Data for LNR and DFS CI: confidence interval; CTRT: chemoradiation; HR: hazard ratio; LNR: lymph node ratio; DFS: disease-free survival

Subgroups		Pooled estimates				Heterogeneity		
		No.of studies	HR	95% CI	P-value	Model	I^2^%	P-value
Overall		14	2.43	2.11-2.80	.00001	Fixed	0%	0.46
No.of nodes	<12	3	2.90	1.85-4.54	.00001	Fixed	0%	0.64
	≥12	13	2.38	2.01-2.80	.00001	Fixed	21%	0.25
CTRT	Yes	14	2.79	2.25-3.47	.00001	Fixed	0%	0.78
	No	3	2.34	1.75-3.15	.00001	Random	48%	0.15
LNR cut-off	0.1	2	1.99	1.58-2.51	.00001	Fixed	0%	0.41
	0.2	6	2.89	2.17-3.84	.00001	Fixed	0%	0.54
	0.3	2	2.35	1.52-3.65	.00001	Fixed	0%	0.77

Publication bias

**Figure 4:**
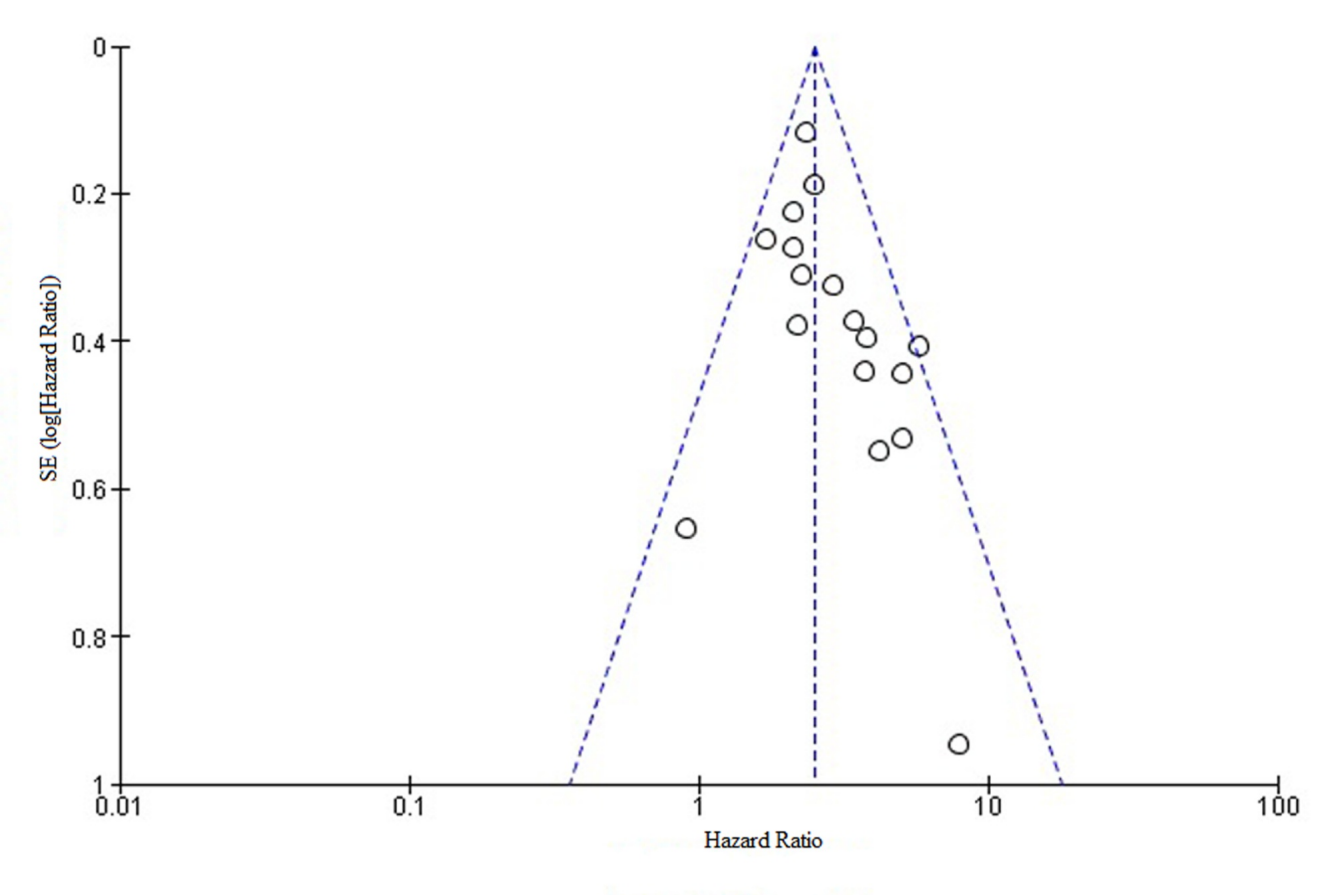
Funnel plot showing LNR and OS OS: overall survival; LNR: lymph node ratio; SE: standard error

**Figure 5:**
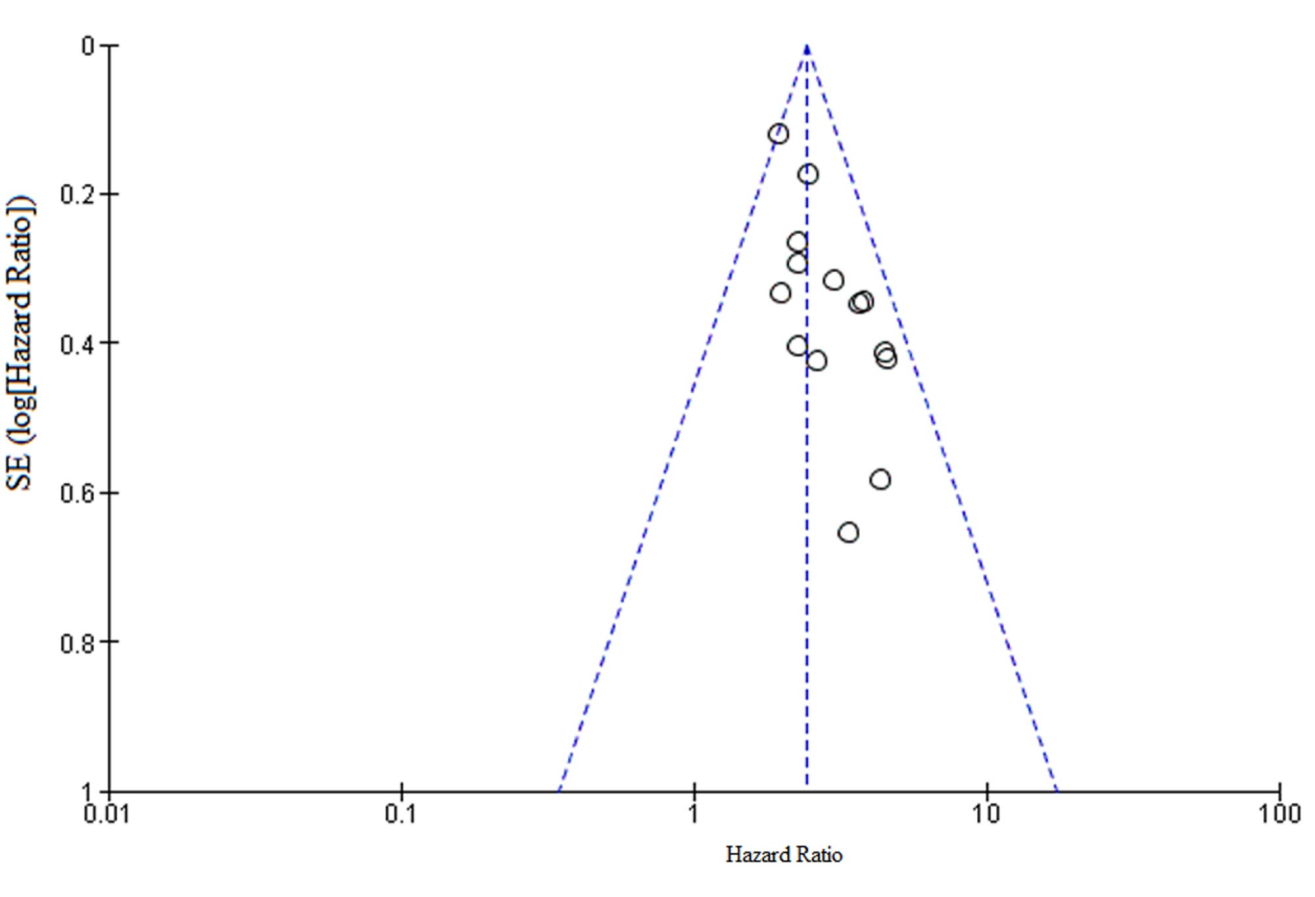
Funnel plot showing LNR and DFS DFS: disease-free survival; LNR: lymph node ratio; SE: standard error

The publication bias of the included studies was evaluated by funnel plots. No visual publication bias was established, as shown in Figure 4 and Figure 5 [6-18,20-23]. This indicated that the publication bias was small in the current meta-analysis.

## Discussion

Lymph nodal metastasis is an essential mechanism involved in the spread of cancers. Quantitative evaluation of the lymph nodal burden has been validated as a powerful prognostic indicator in patients with rectal cancer. Moreover, the absolute number of positive nodes has been recognized as an influential prognostic marker of adverse outcomes. It has been shown that prognosis worsens with the increasing number of metastatic lymph nodes (MLN) [24]. Hence, in the current American Joint Committee on Cancer (AJCC) staging system, the nodal disease is categorized as the N category and is stratified on the basis of the number of MLNs. The N category is further divided into N1 (1-3 MLN) and N2 (>4 MLN) [3]. Although this system has been shown to predict the long-term outcomes with good accuracy, it is well known that TNM does not consider a few other important features regarding lymph node metastasis. Nonetheless, studies have reported that a higher number of negative lymph nodes were independently associated with improved survival in patients with colorectal cancer [25]. To invalidate the limitation of the N stage, LNR has been studied. Our meta-analysis demonstrated the role of LNR in the prognostication of rectal cancer patients. Our pooled results indicate that higher LNR is associated with worse OS and DFS. The finding of low heterogeneity across studies has further added durability to the results.

Fielding et al. suggested that examination of at least 12 lymph nodes as an evaluation of less than that of suggested numbers led to a high false-negative rate of lymph node metastasis and under-staging [26]. The current TNM staging system also recommends the evaluation of 12 lymph nodes to ascertain the proper stage [3]. The number of examined lymph nodes has been reported to be influenced by patient-related factors such as location, stage, and use of neoadjuvant treatment, along with surgical and pathological factors. However, with the emerging interest in the implementation of neoadjuvant chemoradiotherapy (NACRT) and especially its consequent impact on lymph node yield retrieval, controversy still exists regarding the absolute lymph node yield. It is acknowledged that the absolute number of retrieved lymph nodes would significantly reduce with preoperative chemoradiation [27]. This has led to the implementation of LNR to solve the limitations associated with the N category of the TNM staging system.

Some studies have stated that LNR has a significant influence on survival in patients only when the examined rate of lymph nodes is greater than 10-12 [28]. This has raised concern regarding the utility of LNR over the traditional N category of the TNM staging system. Our study results demonstrated that higher LNR is associated with worse OS and DFS and this association remained significant, irrespective of nodal status in rectal cancer patients. These findings were further supported by a recent meta-analysis of 33 studies that included a total of 75,839 patients with node-positive colorectal cancer. In this study by Zhang et al., high LNR was significantly associated with low OS (HR: 1.91; 95% CI: 1.71-2.14; p<.001) and DFS (HR: 2.75; 95% CI: 2.14-3.53; p<.001). They also reported that LNR remained a significant prognostic factor regardless of the number of harvested nodes and reported an HR of 1.97, 95% CI of 1.71-2.26, and p-value of <.001 for the subgroup with more than 12 harvested lymph nodes, and an HR of 1.74, 95% CI of 1.40-2.17, and p-value of <.001 for the subgroup with less than 12 harvested lymph nodes [29].

In the current meta-analysis, we looked for the studies that reported outcomes of patients who underwent NACRT because it has been reported that the total number of retrieved lymph nodes and positive lymph nodes may decrease after chemoradiation [27]. However, our study demonstrated that though neoadjuvant therapy was used in 14 studies, a lymph nodal yield of greater than 12 was observed in 13 studies. Also, our study demonstrated no difference between those with less than 12 lymph nodes and those with more than 12 lymph nodes regarding OS and DFS. These findings are supported by a recent study that demonstrated that though NACRT reduces the lymph nodal yield, it still has no significance on the survival of the patient [30].

The strengths of this meta-analysis are the precision of estimates that are based on a large dataset. This meta-analysis included 18 studies involving 4,486 node-positive rectal cancer patients. The statistical power is satisfactory enough for our results. The other strengths of this meta-analysis are the precision of LNR-specific estimates and the investigation of many covariates. The cut-off value of LNR in each included study is different altogether. The most reliable cut-off value for defining LNR, which could predict the prognosis of rectal cancer patients, is a subject of debate. However, in our study, there is significant statistical power with little heterogeneity when a cut-off of less than 0.2 is examined in a single cut-off stratifying system. However, a large cohort study or an individual patient data meta-analysis is required to justify our results and ascertain infinitesimal differences. Finally, the other strength of our meta-analysis is the minimal heterogeneity between studies and their subgroups, which enhances the robustness of the results.

Our findings should be interpreted within the structure of the effectiveness and limitations of a study-level meta-analysis of heterogeneous studies. There are certain limitations in our study that need to be spelled out. First, there was the inclusion of retrospective studies; therefore, there exists a possibility of unavoidable selection bias. Secondly, in the background of varied cut-off values of LNR generated through various methods, the heterogeneity analysis demonstrated homogeneity. This could have been responsible for likely pooling of these LNR cut-offs in the analysis that was done to predict OS and DFS. And, finally, the surgical and pathological qualities vary among different medical centers in which these studies were conducted.

## Conclusions

Our meta-analysis reviewed the current research targeting the prognostic role of LNR in assessing survival in rectal cancer patients. Our findings have demonstrated that a higher LNR is a predictor of poor OS and DFS. Additionally, our study has demonstrated that LNR is an independent prognostic marker for assessing OS and DFS, irrespective of NACRT and lymph nodal harvest. We conclude that the LNR could provide answers for the lacunae in the N category of the current TNM staging system.
